# THE INTERTISSUE COMMUNICATION BETWEEN THE HYPOTHALAMUS AND WHITE ADIPOSE TISSUE IN MAMMALIAN AGING AND LONGEVITY CONTROL

**DOI:** 10.1093/geroni/igad104.0594

**Published:** 2023-12-21

**Authors:** Shin-ichiro Imai

**Affiliations:** Washington University School of Medicine, St. Louis, Missouri, United States

## Abstract

Recent studies in model organisms have demonstrated that the intertissue communications play a critical role in the regulation of aging and longevity. Our major goal is to understand the systemic regulation of aging and longevity in mammals and translate that knowledge into an effective anti-aging intervention that could make our later lives as healthy and productive as possible (“productive aging”). In mammals, we have previously demonstrated that the intertissue communication between the hypothalamus and adipose tissue, particularly mediated by extracellular vesicles-contained extracellular nicotinamide phosphoribosyltrasferase (eNAMPT), the rate-limiting NAD+ biosynthetic enzyme in mammals, functions to counteract age-associated physiological decline and promote longevity in mice. We have recently found that Ppp1r17-positive neurons in the dorsomedial hypothalamus (DMHPpp1r17 neurons) regulate white adipose tissue (WAT) function, including lipolysis and eNAMPT secretion, through the sympathetic nervous system (SNS), and the feedback loop between DMHPpp1r17 neurons and WAT plays a critical role in the regulation of aging and longevity in mice. Within DMHPpp1r17 neurons, the phosphorylation and subsequent nuclear-cytoplasmic translocation of Ppp1r17, regulated by cGMP-dependent protein kinase (PKG; Prkg1), affect gene expression regulating synaptic function, causing synaptic transmission dysfunction and impaired WAT function. Both DMH-specific Prkg1-knockdown, which suppresses age-associated Ppp1r17 translocation, and the chemogenetic activation of DMHPpp1r17 neurons significantly ameliorate age-associated WAT dysfunction, increase physical activity, decrease age-associated mortality rate, and extend lifespan. Thus, these findings clearly demonstrate the importance of the intertissue communication between the hypothalamus and WAT in mammalian aging and longevity control and provide critical insights into the development of effective anti-aging interventions.

